# Whitebark Pine Stand Condition, Tree Abundance, and Cone Production as Predictors of Visitation by Clark's Nutcracker

**DOI:** 10.1371/journal.pone.0037663

**Published:** 2012-05-25

**Authors:** Lauren E. Barringer, Diana F. Tomback, Michael B. Wunder, Shawn T. McKinney

**Affiliations:** 1 Department of Integrative Biology, University of Colorado Denver, Denver, Colorado, United States of America; 2 National Park Service Inventory and Monitoring Program, Sierra Nevada Network, El Portal, California, United States of America; The Pennsylvania State University, United States of America

## Abstract

**Background:**

Accurately quantifying key interactions between species is important for developing effective recovery strategies for threatened and endangered species. Whitebark pine (*Pinus albicaulis*), a candidate species for listing under the Endangered Species Act, depends on Clark's nutcracker (*Nucifraga columbiana*) for seed dispersal. As whitebark pine succumbs to exotic disease and mountain pine beetles (*Dendroctonus ponderosae*), cone production declines, and nutcrackers visit stands less frequently, reducing the probability of seed dispersal.

**Methodology/Principal Findings:**

We quantified whitebark pine forest structure, health metrics, and the frequency of nutcracker occurrence in national parks within the Northern and Central Rocky Mountains in 2008 and 2009. Forest health characteristics varied between the two regions, with the northern region in overall poorer health. Using these data, we show that a previously published model consistently under-predicts the proportion of survey hours resulting in nutcracker observations at all cone density levels. We present a new statistical model of the relationship between whitebark pine cone production and the probability of Clark's nutcracker occurrence based on combining data from this study and the previous study.

**Conclusions/Significance:**

Our model clarified earlier findings and suggested a lower cone production threshold value for predicting likely visitation by nutcrackers: Although nutcrackers do visit whitebark pine stands with few cones, the probability of visitation increases with increased cone production. We use information theoretics to show that beta regression is a more appropriate statistical framework for modeling the relationship between cone density and proportion of survey time resulting in nutcracker observations. We illustrate how resource managers may apply this model in the process of prioritizing areas for whitebark pine restoration.

## Introduction

Whitebark pine (*Pinus albicaulis*) is a keystone and foundation species of upper subalpine and treeline ecosystems in the western U.S. and Canada [1]–[3]. Whitebark pine is declining nearly rangewide from a combination of white pine blister rust infection (caused by the invasive pathogen *Cronartium ribicola*), mountain pine beetle (*Dendroctonus ponderosae*) outbreaks, and successional replacement from fire suppression [3]. Recently, the species was evaluated as warranting Endangered or Threatened listing and placed with high priority on the candidate species list [4]. Whitebark pine is highly susceptible to blister rust, and only a small to moderate percentage of trees typically show resistance [5]. Mountain pine beetles kill both blister rust-resistant and non-resistant trees, thus reducing the spread of resistant genes. Currently, whitebark pine losses are greatest in the Northern Rocky Mountains of the U.S. and adjacent regions in southern Canada. Blister rust infection levels are high, outbreaks of mountain pine beetle have been rapidly expanding, and fire exclusion leading to successional replacement has reduced the occurrence of whitebark pine as a forest component in these regions over time [3], [6]–[7].

Clark's nutcrackers (*Nucifraga columbiana*) harvest and cache whitebark pine seeds throughout mountainous terrain, typically burying seeds beneath 1 to 3 cm of substrate [8]–[10]. Whitebark pine seed dispersal and seedling establishment almost exclusively results from seed caches that are made but not retrieved by nutcrackers before snow melt and summer rains [8], [10]. However, there exists an asymmetry in the mutualism between whitebark pine and Clark's nutcracker: the pine is an obligate mutualist and primarily depends upon the bird for dispersal of its large, wingless seeds, whereas the bird is a facultative mutualist, able to assess local cone abundance and forage widely on seeds of several *Pinus* species and other food sources [10]. The coevolved, mutualistic relationship between whitebark pine and the Clark's nutcracker is an integral part of the natural history of the Central and Northern Rocky Mountains, as well as other high-mountain regions [10]–[11]. This interaction now appears precarious as whitebark pine succumbs to blister rust and mountain pine beetles. Previous work indicated that nutcrackers are sensitive to the number of seeds available within a stand and are efficient foragers, switching seed resources as cone availability declines [12]. Nutcrackers may be less likely to visit whitebark pine stands with blister rust-diseased trees, which often have fewer cones than healthy trees because of crown damage and tree mortality [13]–[14]. Furthermore, for the last decade, an outbreak of mountain pine beetles in the Rocky Mountains has spread widely and to upper elevations, killing large numbers of whitebark pine [3], [15], and further reducing cone production. With little to no seed dispersal, natural whitebark regeneration is anticipated to decline throughout regions with damaged stands and high mortality. In particular, whitebark pine regeneration in burned areas near these stands may be delayed or greatly reduced. In the Central and Northern Rocky Mountains, whitebark pine occurs as a post-fire pioneer in the upper subalpine zone on productive sites [16].

Predispersal seed predation by North American red squirrels (*Tamiasciurus hudsonicus*) can further constrain whitebark pine regeneration potential. As whitebark pine mortality increases and cone production decreases, red squirrels compete with nutcrackers for whitebark pine seeds and harvest a significant proportion of the cone crop [13], [17]. This predation pressure limits seed dispersal by nutcrackers, and consequently the potential for seedling establishment. McKinney et al. [14] provided the first predictive relationship between estimates of whitebark pine cone production within a stand and the likelihood of nutcracker visitation. The model indicated that no nutcracker visitation occurs when cone production drops below 130 cones/ha.

The historical interactions among squirrels, nutcrackers, and pines have now been altered in many high elevation Rocky Mountain forests, further hastening the decline of whitebark pine [18], [19]. Various researchers have assessed the health of whitebark pine communities throughout the Rocky Mountains within the last 15 years [6], [13], [14], [20]–[22]. Because whitebark pine has historically comprised important ecological communities in the national parks of the Central and Northern Rocky Mountains, the potential loss of this species from anthropogenic factors challenges the mission of the National Park Service “to conserve unimpaired the natural and cultural resources and values of the national park system for the enjoyment of this and future generations.” Furthermore, since the pine is now a candidate species under the Endangered Species Act, forest managers must consider the potential effects of declining cone production capacity on nutcracker habitat use and natural whitebark pine regeneration as they identify areas for restoration treatments. Here, we present data gathered over two field seasons to examine the relationship between whitebark pine community health, cone production, and visitation by Clark's nutcrackers across four national parks in the Central and Northern Rocky Mountains.

### Questions addressed in this study

Overall, we asked whether basal area of live whitebark pine and tree health variables, and thus cone production capacity, could predict the occurrence of nutcrackers in whitebark pine communities. Specific questions addressed include: 1) Do the mean values of whitebark pine live basal area, forest health variables, cone production, and numbers of nutcrackers differ between Northern and Central regions of the Rocky Mountains? 2) Are any of these variables individually more useful for predicting nutcracker occurrence? 3) What is the relationship between cone density and the probability of occurrence of nutcrackers? How well does the relationship that we obtain support a previously published model [14] for predicting the proportion of survey time resulting in bird observations, given cone density? In this study, we use observations of nutcrackers when whitebark pine seeds are ripe as a surrogate or predictor of the likelihood of seed dispersal, a relationship implicit in previous studies [13], [14].

## Methods

### Study areas and transect placement

**Figure 1:**
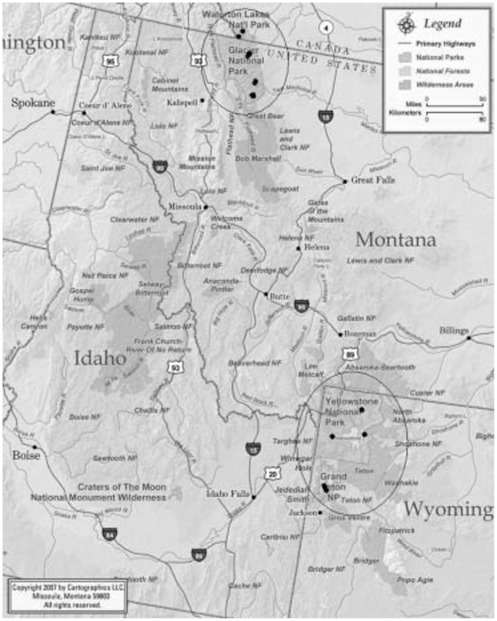
Geographic locations of study sites (solid circles) in four national parks in the southern and northern study regions (open circles), Rocky Mountains. (Map reproduced with permission from Cartographics LLC, www.rockymountainmaps.com).

We selected study sites based on access and geographic representation in Grand Teton and Yellowstone National Parks, Wyoming, USA (Central Rocky Mountains, here referred to as the “southern region”), and in Glacier National Park, Montana, USA, and Waterton Lakes National Park, Alberta, Canada (Northern Rocky Mountains, referred to as the “northern region”) under research permit numbers GRTE-2008-SCI-0025, GRTE-2009-SCI-0042, YELL-2008-SCI-5736, YELL-2009-SCI-5736, GLAC-2008-SCI-0116, and WL-2008-1678, respectively. In July 2008, LEB and DFT established five 1 km×30 m belt transects in the southern region, and another five in the northern region (Fig. 1, Table 1). Transect placement was constrained to enable access by round trip hiking and conducting two point count surveys within one long day, at the request of National Park Service personnel. Consequently, eight transects were associated with established trails, each paralleling a trail approximately 5 m to one side. These trails do not differentiate among various types of whitebark pine habitat; so although transect placement was informed by presence of trails, it is assumed to be random with respect to the distribution of whitebark pine habitat. Two transects in Yellowstone National Park were not associated with trails; one headed upslope cross-country, and the other followed a ridgeline about 25 m from one of the main roads (Table 1). Transects were established by marking trees at 100 m intervals with tree tags and labeled 12 inch nail spikes for the entire total 1 km distance. The start, finish, and pathway of each transect were geo-referenced using a GPS.

**Table 1:** Transect and stand assessment plot descriptions.

Park	Transect	Lat/Lon	Habitat	Elevation (m)	Aspect (°)	Mean DBH (±SD) (cm)	%WBP in Overstory
Grand Teton	Amphitheater Lake	43°43.738 110°46.330	Subalpine forest	2823	20	33.9**±**11.6	39
				2738	185	24.9**±**14.1	38
	Teewinot Mountain	43°44.569 110°45.050	Subalpine forest	2836	5	38.9**±**15.1	57
				2783	5	37.1**±**8.6	40
Yellowstone	Craig Pass	44°25. 564 110°40.060	Subalpine forest	2612	38	14.1**±**12.5	90
				2626	24	8.3**±**12.9	30
	Dunraven Pass	44°47.327 110°26.992	Subalpine meadow and open forest	2868	155	16.9**±**11.4	56
				2801	210	19.3**±**13.3	71
	Avalanche Peak	44°28.697 110°08.070	Subalpine forest	2728	190	36.4**±**17.1	28
				2740	165	23.9**±**8.2	9
Glacier	Siyeh Pass	48°42.819 113°38.813	Subalpine forest	2167	232	33.5**±**10.2	0
				2145	210	25.5**±**8.1	4
	Scenic Point	48°29.112 113°19.074	Subalpine/treeline	2089	210	11.4**±**4.9	29
				2183	210	4.4**±**1.8	73
	Elk Mountain	48°18.137 113°26.566	Subalpine/treeline	2182	220	20.3 (N/A)	25
				2123	219	7.3**±**2.2	21
Waterton Lakes	Summit Lake	49°00.478 114°01.493	Subalpine/open canopy forest	1945	190	1.8 (N/A)	0
				1923	155	15.9**±**13.4	5
	Upper Rowe Lakes	49°03.159 110°03.547	Subalpine/open canopy forest	2182	200	17.4 (N/A)	5
				2196	160	22.7**±**14.6	10

Elevation and aspect were measured at the center of plot (mid-point on plot), and latitude/longitude were recorded from GPS readings taken at the upper end of each transect. Each transect included two rectangular stand assessment plots, 10 m×50 m. Percent whitebark pine in overstory is based on a count of canopy-level trees within each plot. See text for details.

### Stand assessment plots

Stand assessment generally followed an established protocol [23]: Two 10 m×50 m rectangular plots were assessed for each transect to characterize 1) stand structure and composition, 2) diameter at breast height (dbh; “breast height” = 1.37 m height above the ground, measured in cm) for whitebark pine, 3) blister rust infection level (% of living whitebark pine trees infected), 4) percent of whitebark pine trees with mountain pine beetle symptoms, 5) percent of tree mortality and cause, 6) whitebark pine regeneration (number of whitebark pine seedlings), and 7) to count cones. The plots were established at two randomly selected 100 m sections along each transect, with the long dimension of the plot usually parallel, but rarely perpendicular to the transect, depending on topography. If slope steepness or unsuitable habitat excluded use of an area along a transect, a different 100 m section was chosen at random from those that remained. The nail spike marking the selected 100 m section served as one corner of the stand assessment plot. Pin flags in open ground and surveyor's tape in trees were used to demarcate the boundaries of the plot. The start and end points of each plot were geo-referenced with a GPS and marked by tree tags and/or rebar. Once we completed the assessment, transect tapes and pin flags were removed, but notes and photos were taken to assist reestablishment of the identical plot for future sampling.

Within each stand assessment plot, mature cone-bearing canopy-level trees were counted to determine the percentage stand composition by species. Diameter at breast height was recorded for all whitebark pine trees greater than 1 cm dbh on each plot. Diameter was then used to calculate live basal area density (m^2^/ha), here based on the sum over the two 500 m^2^ stand assessment plots. We estimated the mean proportion of the total canopy per tree in each plot that was dead as a result of blister rust damage to branches and consequent foliage loss, newly dead foliage, and mechanical damage. This measurement was categorized into one of the following canopy kill classes for stand description: 1(0–5%), 2(6–15%), 3(16–25%), 4(26–35%), 5(36–45%), 6(46–55%), 7(56–65%), 8(66–75%), 9(76–85%), 10(86–95%), 11(96–100%). Secondary blister rust infection symptoms (e.g., branches with red-brown foliage, sap oozing, and rodent gnawing) were noted, but only live trees with active (i.e., with old or new aecial sacs) or inactive cankers were classified as infected with blister rust [23]. Mountain pine beetle attack on a tree was indicated by entry holes with or without pitch tubes in the boles of trees, and/or recent emergence holes; recently attacked trees had green foliage, and trees attacked in the previous one or two years were indicated by foliage fading over the canopy from green to red-brown. All trees with >1% green foliage were still classified as “living” regardless of condition. J-shaped adult beetle galleries and horizontal larval galleries engraved in exposed wood were used to identify trees killed by mountain pine beetles in previous decades.

We recorded the cause of all whitebark mortality where discernible. Dead trees were counted and dbh measured even if the cause of death could not be determined. We updated records of mountain pine beetle attacks in late summer 2008, and again during early and late summer in 2009. Whitebark pine regeneration was quantified by methodically searching both stand assessment plots associated with each transect and counting all seedlings ≤50 cm in height within each plot.

### Cone, nutcracker and squirrel counts

In 2008 and 2009, we counted whitebark pine cones in each stand assessment plot by standing about 5 to 10 m from a tree and using binoculars to search the canopy. All whitebark pine trees were examined for cone production. A minimum of two people counted cones from different vantage points, and the average value was recorded. We counted cones in each plot twice per summer: first in mid-late July, before nutcrackers disperse seeds and squirrels cut down cones, and again between late August and early September, after seed dispersal is underway. Counts may either increase or decrease across summer for the following reasons: When spring temperatures are cold and cone maturation is delayed, early cone counts may miss cones resulting in a larger value for the second count. Squirrels may cut down cones between the first and second counts resulting in a lower value for the second count. We used the larger of these two numbers (first and second cone counts) for each plot to compute cone density by summing the counts for the two plots in a given transect to give a total number of cones per 1000 m^2^; this number was then multiplied by a factor of 10 to estimate the number of cones per hectare.

To standardize nutcracker counting, we established, marked, and geo-referenced six point count stations, one every 200 m (starting at 0 m), along each transect. Because the point counts were primarily for inventory, we recorded the number of nutcracker detections for ten minutes at each point count station. Data collected during each point count included start time and end time, number of nutcrackers observed, nutcracker vocalizations without sightings, and also number of red squirrels observed or heard. Nutcrackers heard nearby but not sighted during point counts were classified as an observation. We attempted to avoid counting the same nutcrackers twice by noting nutcracker movements whenever possible. When nutcrackers could only be heard, we followed their call directions in order to count them only once. Each point count station was visited twice per summer, at the same time that cones were counted. Point counts were taken twice per day on each visit; one set of points was visited between 08:00 and 10:00 am and then another set between 1:00 and 6:00 pm, with at least a 2.5-hour window between counts. Thus, during each summer, a total of 240 min of time was spent gathering observations for each transect.

### Data analysis

We carried out two distinct statistical analyses of our data. First, we used MANOVA to describe differences in population means for each of the stand assessment variables, and squirrel and nutcracker counts between years and regions; the goal of this analysis was to determine whether there were any non-random differences between years or locations. Explanatory variables, therefore, included region, year, and interaction of region and year. Response variables included number of live, healthy whitebark pine (WBP) trees; percent canopy kill for WBP; proportion of live WBP trees with blister rust infection; proportion of WBP trees with pine beetle infestation; numbers of WBP seedlings; number of cones counted in WBP; live basal area of WBP; total basal area of WBP; total number of squirrel observations; and the sum of nutcracker counts in 2008 and 2009.

Second, we used logistic regression in an exploratory analysis to determine the relative weight of support for each of nine independent variables as predictors for the probability of nutcracker occurrence; the goal of this second analysis was to identify any potentially useful covariates for predicting the probability of nutcracker occurrence in tree stands. We fit logistic regression models for all 512 possible combinations of the nine explanatory variables, including an intercept only model, in order to identify the relative importance weights from Akaike Information Criterion statistics adjusted for small sample sizes (AIC_c_) for each of the variables [24]. We computed AIC_c_ weights to compare models using
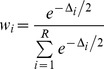
(1)where R is the number of models in the set and Δ_i_ is the difference between the AIC_c_ score for model i and the lowest overall AIC_c_ score for the models in the set. The ratio of AIC_c_ weights (w_i_/w_j_) for any two models is called an evidence ratio, and it quantifies the relative degree of support in the data for one model as compared to another [24]. For comparative reference only, we also present the subset of models that were within two AIC_c_ units of the model with the lowest AIC_c_ value as the set of most parsimonious models in the exploratory analysis [24].

### Cone threshold model evaluation

McKinney et al. [14] predicted nutcracker occurrence based on an index of cone production using a linear regression parameterized as y = −0.449+0.019×, where y is the proportion of observation hours that resulted in observation of one or more nutcrackers, and x is (ln(cones/ha))^2^. The ln-transform was used to normalize the distribution of cone densities. We used our data as an independent test of this model. To accomplish this, we converted our data for the number of nutcracker observations per point count transect to the proportion of observation hours resulting in at least one nutcracker observation, and transformed our cone densities to the same index of cone production used by [14]. We compared the 2008 and 2009 observed values for proportion of observation hours resulting in nutcrackers with values predicted from [14] using the observed cone densities from 2008 and 2009 in this study. We fit a linear regression model to the new data to compare parameter estimates based on the new data with those given by [14]. Because the response variable for these models is a proportion, we also fit a regression using a logit link and a beta-distributed error term [25] to the new data, to the data from [14], and to the combined data sets of the two studies. We computed AIC_c_ weights as described previously to compare the efficacy of the linear and beta regression for each of the three data sets just described. We used R version 2.10.1 to perform all statistical analyses.

## Results

### Forest conditions and numbers of nutcrackers and squirrels

**Figure 2:**
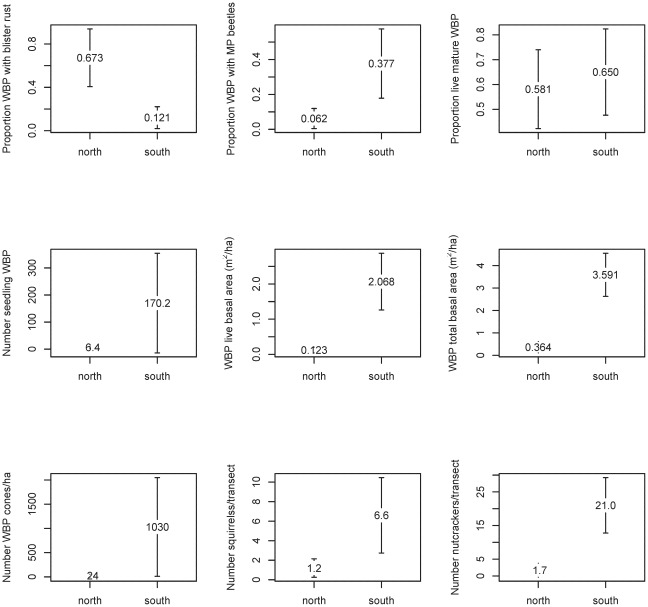
Mean values of stand assessment variables, cone number, and squirrel and nutcracker counts for each region. Data are summed from two stand assessment plots per transect and averaged across transects. Error bars indicate extent of 95% confidence intervals around the means. Abbreviations: WPB whitebark pine, MP mountain pine beetle.

The pattern of differences in regional means examined by the MANOVA for the nine measured variables was the same across years (there was no significant interaction effect between year and region, *P* = 0.76; Fig. 2). There was no significant difference in means between years when pooling over region (*P* = 0.65), but there was a significant difference between means by region when pooling over years (*P* = 1.9×10^−5^). Collectively, these results suggest that space, and not time (two different years), had the greatest influence on forest health conditions and use by squirrels and nutcrackers (Fig. 2, Appendix S1, S2).

Generally, the northern region was characterized by having a higher proportion of whitebark pine trees infected by blister rust than in the south. In contrast, the proportion of whitebark pine trees attacked by mountain pine beetle was higher in the southern region than in the north (Fig. 2). Although the proportion of live mature whitebark pine trees was similar for both regions, both the number of seedlings (regeneration) and the average cone density on stand assessment plots were higher in the southern region than in the north; however, the southern region was also much more variable in terms of both regeneration and cone density than was the northern region (Fig. 2). The total basal area occupied by whitebark pine was greater in the southern region than in the north, and the southern region also supported higher average counts of both squirrels and nutcrackers (Fig. 2).

Cone densities were positively correlated with live basal area (*r* = 0.55) and with proportion of observation hours resulting in nutcracker observations (*r* = 0.75). We observed nutcrackers in stands with mean live whitebark pine basal area of 1.5±0.09 m^2^/ha (mean ± s.e.m.; range = 0.04–3.23 m^2^/ha; *n* = 14). The mean live basal area for stands where no nutcrackers were observed was 0.1±0.02 m^2^/ha (mean ± s.e.m.; range = 0.04–0.33 m^2^/ha; *n* = 6).

When compared to the northern region, the stand assessment plots in the southern region had a significantly higher percentage of live whitebark pine basal area (*P* = 0.0001) and a significantly higher combined live and dead basal area, or total basal area (*P* = 3.4×10^−6^); overall, live basal area and total basal area for whitebark pine trees were much lower in the northern region (0.364 m^2^) than in the southern (3.591 m^2^, Fig. 2, Appendix S1). The northern region had a higher proportion of blister rust infection (*P* = 0.0007), a lower proportion of mountain pine beetle infestation (P = 0.005), fewer whitebark pine seedlings (*P* = 0.07), lower whitebark pine cone density (*P* = 0.03), fewer encountered red squirrels (*P* = 0.01), and fewer encountered nutcrackers (*P* = 0.0002; Fig. 2, Appendix S1). The proportion of whitebark pine trees that were living was similar for both regions (0.581 for the north vs. 0.650 for the south, *P* = 0.54; Fig. 2). In general, the overhead canopies of stands surveyed in the southern region contained more live whitebark pine than in the northern region (Table 1, Appendix S1).

### Predicting nutcracker occurrence

No single variable emerged from the logistic regression models as a comparatively superior predictor of the probability of nutcracker occurrence (Table 2). Although the live proportion of mature whitebark pine trees was slightly more important for predicting the probability of nutcracker occurrence, the overall distribution of variable importance weights was essentially uniform (Table 2). The most parsimonious model included percent living whitebark pine, geographical region, and number of red squirrels. On the logit scale, the coefficients for this model were intercept = −120.12, percent living whitebark pine = 232.46, number of squirrels = −21.96, and region.south = 353.32. None of the remaining 511 models were within 2 AIC_c_ units of this top model.

**Table 2:** Variable Importance Weights.

Variables	Proportion of weights among variables
Proportion live mature WBP	0.12
Proportion WBP with blister rust	0.11
Proportion WBP with pine beetle	0.11
Number of seedling WBP	0.11
Number of WBP cones	0.11
Basal area of live WBP	0.11
Basal area of live+dead WBP	0.11
Number of squirrels	0.11
Geographic region	0.11

AIC-based variable importance weights for predictive variables in logistic regression models for predicting the probability of nutcracker occurrence.

### Cone threshold model comparison

**Figure 3:**
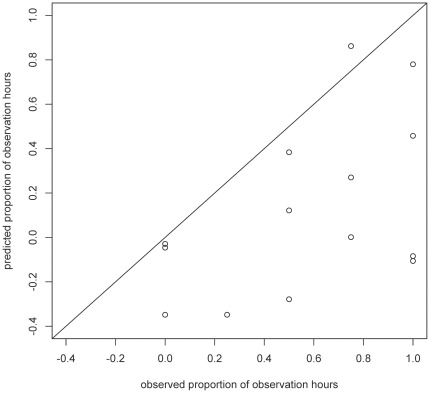
Observed values for the proportion of observation hours resulting in nutcracker visitation in 2008 and 2009 versus that predicted from cone production values using the model presented by McKinney et al. [**14**]. The diagonal is the 1∶1 line that represents perfect prediction from the model. All but one observation occurs below the 1∶1 line, indicating that the McKinney et al. model consistently under-predicted the probability of nutcracker occurrence for the cone production values observed in this study.

Our 2008 and 2009 data generally indicated a lower cone threshold for nutcracker visitation than did the model in [14]: in all but one case for the new data, the earlier model [14] under-predicted the proportion of observation hours resulting in nutcracker sightings, based on the same cone production index (Fig. 3). The estimated intercept for a linear regression fit to the new data was 0.178; the 95% confidence interval around this estimate (−0.042, 0.397) does not include −0.449, the intercept estimated by [14]. Further, the 95% confidence interval around the intercept estimated for the data published by [14] was (−0.555, −0.321), a range that does not include 0.178, the estimate for the intercept based on the new data. The slope estimate for the linear regression line fit to the new data was 0.012, a value that was significantly different from that published by [14]. The 95% confidence interval around this new estimate (0.005, 0.019) barely includes 0.019, the slope reported by [14]; the 95% confidence interval around the slope estimate for the data in [14] was (0.015, 0.024), a range that does not include the 0.012 estimate from fitting a linear model to the new data.

Beta regression models were far more parsimonious with the data than were the linear regressions discussed above. For the new data, the AIC_c_ for the linear regression was 13.3 as compared with −16.3 for a beta regression model based on the same data. Likewise, the AIC_c_ for the linear regression fit of the data published in [14] was −5.9 as compared with −39.0 for the beta regression of on the same data. When we pooled the two datasets, we found the beta regression to be even more parsimonious (AIC_c_ for the linear model was 20.9 as compared with −46.7 for the beta regression). The Akaike weights for the data collected in this study were ∼1.0 for the beta regression and 3.7×10^−7^ for the linear regression, meaning that the beta regression was 2.7×10^6^ times more likely as the better model for the data than was the linear regression. For the data in [14], the weights were ∼1.0 for the beta regression and 6.5×10^−8^ for the linear model, indicating that the beta regression was 1.5×10^7^ times more likely than the linear model as the most parsimonious model for the data. A similar comparison was observed when pooling the data sets, with ∼1.0 for the beta regression and 2.1×10^−16^ for the linear regression, indicating that the beta regression was 4.8×10^14^ times more likely to be the better model for the combined datasets. In other words, the beta regression model was far more parsimonious than the linear model for the data collected in this study, for the data presented in [14], and for the two datasets combined. In addition, the linear model determined by [14] generated out-of-sample predictions (i.e., when transformed cone density was <26, the model predicted negative proportions of hours resulting in bird observations), whereas the logit link in the beta regression model does not, by definition, produce out-of-sample predictions for [0,1] random variables.

## Discussion

For more than a century, blister rust has been increasing across the range of whitebark pine [3]. Until recently, Waterton Lakes and Glacier National Park in the Northern Rocky Mountains and Yellowstone and Grand Teton National Park in the Central Rocky Mountains represented two extremes with respect to blister rust impact. Recent assessments in Glacier and Waterton Lakes National Parks indicated that mean blister rust infection levels were 67% and 71.5%, respectively [22], whereas recent assessments in the Greater Yellowstone Area indicated regional means of 24.9% or 39.8%, depending on plot subset [26]. Furthermore, mountain pine beetle outbreaks now range from British Columbia to California and east throughout the Rocky Mountains, killing large numbers of whitebark pine especially in the Greater Yellowstone Area, but also in the northwestern U.S., and western Canada [15].

Whitebark pine damage and mortality from blister rust coupled with widespread losses from mountain pine beetle have drastically affected whitebark pine health and abundance, reducing seed availability for Clark's nutcrackers. Earlier observations [13]–[14] and those in this study indicate that reduced seed crops are already resulting in reduced visitation of whitebark pine communities by nutcrackers.

### Variation in whitebark pine community health factors

Live basal area and total basal area for whitebark pine trees were much lower in the northern region than in the southern (Fig. 2, Appendix S1), indicating that there are more whitebark pine trees in high elevation forests of the southern region of the study. Although some differences in whitebark pine density between the regions may in part result from topographic and climatic factors, whitebark pine has diminished in the Northern Rockies over decades from previous mountain pine beetle outbreaks, successional replacement, and high infection levels of blister rust [6], [14], [22]. Both mean whitebark pine cone density and whitebark pine regeneration were much more variable in the southern portion of the study area than in the north; but, on average, regeneration in the southern region was about 26 times greater than in the north, and the mean cone density was about 43 times greater in the south than that in the north (Fig. 2). Blister rust infection rates were significantly lower in the southern region as compared with the northern portion of the study area, while pine beetle infestation rates were significantly higher in the southern as compared with the northern region (Fig. 2). Taken together, these results demonstrate that the whitebark pine forests are more expansive in the southern portion of our study area than in the northern portion. Not surprisingly then, both the average number of squirrels observed per transect and the average number of Clark's nutcrackers observed per transect were significantly higher in the southern region of the study area than in the north (Fig. 2).

### Predictors of nutcracker visitation

Of the variables we measured and tested for predictive value, geographic region, number of red squirrels, and the proportion of whitebark pine trees that were living all appeared in the most parsimonious model. Clark's nutcrackers were observed far more often in the southern region than in the northern region, where there were more live whitebark pine trees, as indicated by the higher live basal area and proportion of live trees (Fig. 2, Appendix S2). Although the southern region also had a higher mean number of cones (Fig. 2), the distributions were variable enough to obscure any population-level differences between the northern and southern regions. Thus, the proportion of whitebark pine trees that were living was a better indicator of visitation by nutcrackers, where an increase in the proportion of live trees increased the odds of visitation by nutcrackers, although only slightly (Table 2), and the number of red squirrels was also associated with the top model for predicting nutcracker visitation, suggesting use of healthy whitebark pine trees by both species.

### Application and management implications of predictive model

As whitebark pine damage and mortality increase within a stand, cone production diminishes, and the probability of nutcrackers dispersing whitebark pine seeds declines. Restoration strategies are currently being devised or implemented for many western national forests and national parks [3], [7]; for example, planting blister rust-resistant seedlings where cone production has been diminished, but also reducing the density of shade-tolerant competitors and using prescribed fire to encourage nutcracker seed caching. The ability to predict the likelihood of nutcracker seed dispersal within a forest, or across a given landscape, based on cone production estimates, provides a tool for helping to prioritize forest communities for restoration treatments.

**Figure 4:**
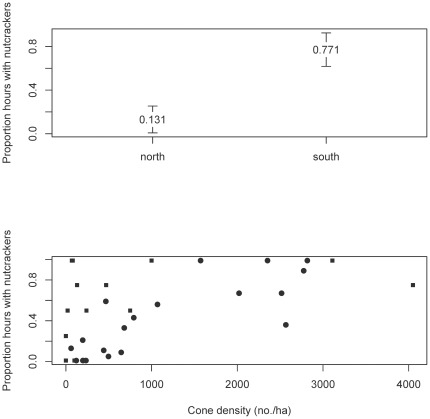
Upper panel: Average number of Clark's nutcrackers observed per transect in the two study area regions. Error bars indicate extent of 95% confidence intervals. Lower panel: proportion of observation hours resulting in an observation of Clark's nutcracker as related to average density of whitebark pine cones on survey plots sampled in this study (squares) combined with those sampled in [14] (circles).

Our data combined with those collected by [14] suggest that whitebark pine communities are regularly surveyed or “cruised” by Clark's nutcrackers in search of cones, and as cones are discovered, nutcrackers are more likely to be present. As cone density in a given forest stand increases, so too does the likelihood that an observer will detect a nutcracker in the stand, but nutcrackers still visit stands that have very few cones (Fig. 4). Previous observations of nutcracker exploration of different seed sources to assess food availability support these findings [12]. In this study, we observed nutcracker visitation in stands with cone densities ranging from 0–4,050 cones/ha. However, the proportion of observation hours resulting in nutcracker observations was reliably above ∼0.75 for cone densities of 1000/ha and above (Fig. 4). In this study, these densities were associated with live whitebark pine basal areas of ≥2.0 m^2^/ha. These results are largely consistent with earlier findings, although the threshold for live basal area is somewhat lower than reported in the previous study [14].

The beta regression model coefficients ± standard errors for the data published by [14] on the logit scale were −4.6±0.70 (intercept) and 0.10±0.02 (slope). For the data collected in this study, they were −1.05±0.44 (intercept) and 0.04±0.01 (slope). For the two datasets pooled together, the estimates were −1.52±0.37 (intercept) and 0.04±0.01 (slope). Exponentiating the slope coefficient from the models describes how the odds of observing nutcrackers change on average as a function of the cone density index. For the data in [14], the odds increase by about 11% for every unit change in the cone density index. For the data collected in this study, and for the two datasets pooled, the odds of observing nutcrackers increases by about 4% for every unit change in cone density index. Exponentiating the intercept coefficient from the models describes the on-average odds of observing nutcrackers when there are no whitebark pine cones present. For the data in [14], the odds were about 1∶99 (0.01) of observing nutcrackers vs. not observing them when there are no cones present. For the data collected in this study, the odds were 1∶2 (0.35) of observing nutcrackers vs. not observing them when there are no cones present. For the two datasets pooled, the odds of observing nutcrackers vs. not observing them when no cones are present was about 1∶4 (0.22).

Using the beta regression model for the pooled datasets (i.e., the most complete information), we can compute the cone density for any given odds ratio (probability) of observing nutcrackers. For example, a reasonable threshold value for cone density might be that where the odds are 50∶50 for observing a nutcracker. The parameterized beta regression model for the pooled datasets is 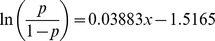 where *p* is the probability of observing nutcrackers and *x* is the cone density index (ln(cones/ha))^2^. Solving the model for *x* when *p* = 0.5 gives 
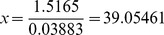
. Converting this number from the cone density index to raw cone density in cones/ha is 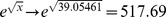, or about 518 cones/ha. This model can be applied in reverse as well: if one wants to compute the probability of observing nutcrackers in a particular tree stand, given a specific cone density, that can be computed as 
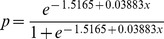
 where *x* is the cone density index (ln(cones/ha))^2^. For example, to compute the probability of observing nutcrackers given the threshold cone density value of 130 cones/ha as proposed by [14], this is done by first converting the density from units cones/ha to the squared log scale and then plugging into the logit transform as follows: x = (ln(130))^2^ = 23.69 and then 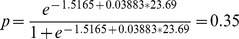 indicating that there is roughly a 35% chance of observing a nutcracker in the stand when the cone density is at this level.

Because this conclusion differs in part from the one presented in [14], some discussion of three potential explanations is warranted. It is possible that different sampling methods may have led to this model predicting a lower cone threshold than that in [14]. In this study, we estimated cone density by counting cones on all trees within a 1000 m^2^ sample area using binoculars. In [14], a spotting scope was used to count cones on 2–4 trees that were known to have cones. The mean number of cones was calculated in [14] by counting cones on two to four cone trees per 1-ha block, with each site consisting of two to seven hectares (thus four to 28 cone trees per site). The mean cone value was multiplied by the number of cone-bearing trees that were counted within two 1000 m^2^ plots within each 1 ha block (thus, 4,000 to 14,000 m^2^). Therefore, cone density in our current study may have been biased low if any cones were missed, and density in the earlier study may have been biased high if the trees that were chosen for counting had a greater than average number of cones. Second, [14] searched 1 ha blocks for nutcrackers, whereas we surveyed points along a 1 km transect in this study. Thus, the current study recorded nutcrackers in an area roughly 10-fold the size of the 1-ha sample unit used in [14]. Unfortunately, neither study quantified the probability of detection for cones or for birds, so we cannot objectively evaluate the merit of these potential explanations.

Another explanation for the general differences in parameter values between the two study's models is that nutcrackers do not restrict their cone searching to 0.1 ha plots, the plot size that we sampled for cones; the typical home range for nutcrackers during summer may be on the order of 100–300 ha [27] and individual birds may be relatively long-lived [10]. So, because nutcrackers are long-lived and range widely, it is possible for an individual nutcracker to visit multiple stands within a larger landscape each year, and a greater region within a lifetime. We therefore suggest that monitoring of whitebark pine forests for cone density is better done at a landscape level, rather than at the stand level. For example, if there is a strong relationship between remotely sensed values of whitebark pine live canopy and ground-surveyed cone abundance, one could use imagery to calculate cone density across a larger area (e.g. ∼1000 ha) than can be accomplished on the ground. Until landscape level approaches to monitoring are developed, however, we encourage practitioners to use stand level data and the parameterized beta regression model in this paper to help monitor and forecast potential interactions between cone production and stand visitation by Clark's nutcrackers, and to plan restoration treatments; for example, landscapes with whitebark pine cone production corresponding to high probabilities of seed dispersal are more likely to regenerate naturally. This information may be used in planning and prioritizing restoration activities [3], [14]. Although the model presented here is based on the most comprehensive data available, and represents a clear improvement on earlier models, it is still only our best estimate to date for relating nutcracker visitation rates as a function of cone production.

### Conclusions

Far fewer living whitebark pine trees and fewer Clark's nutcrackers were observed in the northern study areas than in the southern; less tree regeneration was also observed in the northern region. Without the implementation of restoration efforts, the few remaining healthy whitebark pine stands in the Northern Rocky Mountains will likely continue to decline from the combination of high infection incidence of *Cronartium ribicola*, historical and current losses of whitebark pine from mountain pine beetle infestation, and successional replacement of whitebark pine by more shade-tolerant trees, thus reducing cone production and potentially disrupting the nutcracker-whitebark pine mutualism in this region. This could lead to further fragmentation loss of seed dispersal services, and, eventually, the extirpation of whitebark pine in this region [3], [18]. The model presented here can help managers prioritize whitebark pine communities for restoration efforts, by enabling them to predict nutcracker visitation from cone production estimates.

## Supporting Information

Appendix S1 **Transect health plot variables.** Percentages, canopy kill class, and DBH were based on means of both stand assessment plots per transect; and, LBA measurements, total dead, and regeneration were based on sums across both stand assessment plots of a transect. See Table 1 for study site abbreviations.
(DOCX)

Appendix S2 **Cone counts summed across stand assessment plots for each study site, and counts for nutcrackers, and squirrels.** See Table 1 for park and study site abbreviations.
(DOCX)
