# Supplementary material for: Whitebark Pine Stand Condition, Tree Abundance, and Cone Production as Predictors of Visitation by Clark's Nutcracker
Source: PLoS One. 2012 May 25;7(5):e37663. doi: 10.1371/journal.pone.0037663 (PMC3360761; doi:10.1371/journal.pone.0037663)
Supplement: Appendix S1 — Transect health plot variables. Percentages, canopy kill class, and DBH were based on means of both stand assessment plots per transect; and, LBA measurements, total dead, and regeneration were based on sums across both stand assessment plots of a transect. See Table 1 for study site abbreviations. (DOCX) [file pone.0037663.s001.docx]

Appendix S1. Transect health plot variables. Percentages, canopy kill class, and DBH were based on means of both health plots per transect; and, LBA measurements, total dead, and regeneration were based on sums across both health plots of a transect. See Table 1 for study site abbreviations.

|  | **Park** | | | | | | | | | |
| --- | --- | --- | --- | --- | --- | --- | --- | --- | --- | --- |
|  | **GTNP** | | **YNP** | | | **GNP** | | | **WLNP** | |
| **Transect** | **AL** | **TM** | **CP** | **DP** | **AP** | **SP** | **SCP** | **EM** | **SL** | **RL** |
| DBH (cm) min-max  avg,  median | 2-51 27.6  29.4 | 3-63  37.0  38.3 | 1-64 11.5  5.7 | 1-47  18  17 | 8-82 33.2  32 | 17-45  30.0  32.1 | 2-23  17.0  6.5 | 5-20  8.6  7.6 | 2-3  13.0  8.2 | 1-50  22.1  21.9 |
| Percent of live trees with blister rust (2008, 2009 were the same value) | 32.1 | 24.0 | 1.1 | 3.1 | 0.0 | 0.0 | 70.6 | 100.0 | 75.0 | 90.9 |
| Percent of all trees killed by pine beetle (2008/2009)***** | 27.5 | 40.9 | 0.0 | 28.4 | 82.6 | 0 | 0 | 0 | 20.0 | 83.3 |
|  | 45.0 | 52.3 |  |  |  |  |  |  |  |  |
| Canopy kill class (avg) | 3.7 | 3.6 | 1.1 | 1.3 | 1.6 | 2.0 | 5.5 | 2.5 | 8.6 | 4.1 |
| Total LBA for both plots combined and for m^2^/ha | 1.826  18.26 | 3.228  32.28 | 2.029  20.29 | 3.029  30.29 | 0.2289  2.29 | 0.0821  0.821 | 0.112  1.128 | 0.0436  0.436 | 0.0421  0.421 | 0.3561  3.561 |
| Total number of dead WBP trees | 12 | 19 | 5 | 29 | 17 | 5 | 4 | 6 | 1 | 7 |
| Regeneration *** | 9 | 26 | 645 | 160 | 11 | 3 | 17 | 4 | 2 | 6 |
| *****Mountain pine beetle infestation levels increased in Grand Teton NP from 2008 to 2009. It was more consistent in the other parks.  ****** Canopy kill classes: 1(0-5%), 2(6-15%), 3(16-25%), 4(26-35%), 5(36-45%), 6(46-55%), 7(56-65%), 8(66-75%), 9(76-85%), 10(86-95%), 11(96-100%)  *******Regeneration, defined as number of seedlings ≤ 50 cm height, was summed across both health subplots | | | | | | | | | | |
