# Supplementary material for: Whitebark Pine Stand Condition, Tree Abundance, and Cone Production as Predictors of Visitation by Clark's Nutcracker
Source: PLoS One. 2012 May 25;7(5):e37663. doi: 10.1371/journal.pone.0037663 (PMC3360761; doi:10.1371/journal.pone.0037663)
Supplement: Appendix S2 — Cone counts summed across stand assessment plots for each study site, and counts for nutcrackers, and squirrels. See Table 1 for park and study site abbreviations. (DOCX) [file pone.0037663.s002.docx]

Appendix B. Cone counts summed across health plots for each study site, and counts for nutcrackers, and squirrels. See Table 1 for park and study site abbreviations.

| **Park** | | **GTNP** | | **YNP** | | | **GNP** | | | **WLNP** | |
| --- | --- | --- | --- | --- | --- | --- | --- | --- | --- | --- | --- |
| **Study site** | | **AL** | **TM** | **CP** | **DP** | **AP** | **SP** | **SCP** | **EM** | **SL** | **RL** |
| Cone counts | 2008 | 13 | 7 | 47 | 138 | 2 | 10 | 3 | 0 | 0 | 0 |
|  | 2009 | 24 | 75 | 405 | 311 | 8 | 11 | 0 | 0 | 0 | 0 |
| Nutcracker counts* | 2008 | 10 | 31 | 26 | 30 | 7 | 0 | 6 | 0 | 2 | 1 |
|  | 2009 | 18 | 18 | 44 | 13 | 13 | 0 | 8 | 0 | 0 | 0 |
| Squirrel counts* | 2008 | 14 | 10 | 6 | 4 | 1 | 0 | 1 | 0 | 2 | 0 |
|  | 2009 | 17 | 6 | 4 | 2 | 2 | 1 | 2 | 0 | 4 | 2 |
| * Observations are summed for all point counts per year | | | | | | | | | | | |
